# Promising interlayer sensitization strategy for the construction of high-performance blue hyperfluorescence OLEDs

**DOI:** 10.1038/s41377-024-01490-6

**Published:** 2024-06-13

**Authors:** Jianghui Wang, Peng Zou, Letian Chen, Zhentao Bai, Hao Liu, Wen-Cheng Chen, Yanping Huo, Ben Zhong Tang, Zujin Zhao

**Affiliations:** 1 State Key Laboratory of Luminescent Materials and Devices, Guangdong Provincial Key Laboratory of Luminescence from Molecular Aggregates, South China University of Technology, Guangzhou, 510640 China; 2 School of Chemical Engineering and Light Industry, Guangdong University of Technology, Guangzhou, 510006 China; 3 School of Science and Engineering, Shenzhen Institute of Aggregate Science and Technology, The Chinese University of Hong Kong, Shenzhen, Guangdong 518172 China

**Keywords:** Organic LEDs, Photonic devices

## Abstract

Multi-resonance thermally activated delayed fluorescence (MR-TADF) materials are promising candidates for organic light-emitting diodes (OLEDs) with narrow electroluminescence (EL) spectra. Current researches focus on fabricating hyperfluorescence OLEDs to improve EL efficiencies of MR-TADF emitters by co-doping them with TADF sensitizers in a single host layer. However, in many cases, the polarity of the single host could be not suitable for both blue MR-TADF emitters and blue TADF sensitizers, resulting in broadened EL spectra in high-polar hosts or decreased EL efficiencies in low-polar hosts. Herein, we wish to report an efficient sensitization strategy for blue MR-TADF emitters by constructing an interlayer-sensitizing configuration, in which the blue TADF sensitizers and blue MR-TADF emitters are separated into two closely aligned host layers with high polarity and low polarity, respectively. Based on this strategy, efficient blue hyperfluorescence OLEDs are realized and verified by employing various TADF sensitizers and different MR-TADF emitters, furnishing outstanding external quantum efficiencies of up to 38.8% and narrow EL spectra. These results validate the feasibility and universality of this interlayer sensitization strategy, which provides an effective alternative to high-performance blue hyperfluorescence OLEDs.

## Abstract (web summary)

A promising sensitization strategy is proposed for blue MR-TADF emitters by constructing interlayer-sensitizing configuration, and high-performance blue hyperfluorescence OLEDs with high EQEs and narrow EL spectra are realized.

## Graphical abstract

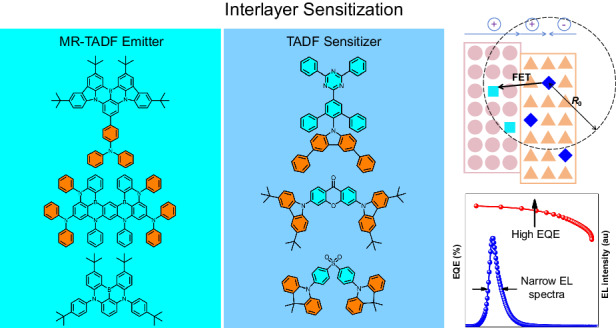

## Introduction

High-quality organic light-emitting diode (OLED) displays require high efficiency, high brightness, high stability, and high color purity simultaneously^1–4^. With the light-emitting materials of OLEDs developing to the third generation of purely organic thermally activated delayed fluorescence (TADF) materials, the efficiency, brightness, and stability have been greatly improved, and some parameters have fulfilled the requirements for the commercialization of OLEDs^5–8^. However, due to the strong intramolecular charge transfer (CT) effect, normal TADF materials featuring a twisted donor‒acceptor (D‒A) configuration exhibit broad emission spectra, which makes it difficult for them to acquire high color purity^9–14^.

Thanks to the advent of multi-resonance TADF (MR-TADF) materials, it becomes realizable readily to fabricate high-performance OLEDs with superb color purity^15–17^. In MR-TADF molecules, the electron density populations of the highest occupied molecular orbitals (HOMOs) and the lowest unoccupied molecular orbitals (LUMOs) are alternately distributed on the atoms due to the complementary resonance effect. Such kind of frontier molecular orbital distributions in a rigid molecular framework can minimize vibronic coupling and vibrational relaxation of the excited states, leading to a series of extraordinary photophysical properties such as high photoluminescence quantum yields (PLQY or *Φ*_PL_), fast radiative decay, and narrow full-width at half-maximum (FWHM) of emission spectra^18–21^. Therefore, MR-TADF materials are promising candidates to improve the color quality of OLEDs.

Although MR-TADF molecules show TADF characteristics, they usually have relatively large energy splitting (Δ*E*_ST_) between the lowest excited singlet (S_1_) state and the lowest excited triplet state (T_1_), which leads to slow reverse intersystem crossing (RISC) and thus long delayed fluorescence (DF) lifetime^22,23^. Consequently, many MR-TADF molecules suffer from serious triplet-involved quenching processes, which undermine electroluminescence (EL) efficiencies and cause large efficiency roll-offs at high luminance. Many current researches focus on addressing this issue by fabricating hyperfluorescence devices, in which conventional TADF sensitizers with short DF lifetimes are incorporated to sensitize MR-TADF molecules^24–29^. So far, the EL performances of many MR-TADF molecules have been successfully improved by this approach. But for blue MR-TADF molecules, the current sensitization configuration of co-doping MR-TADF emitters with TADF sensitizers in the same wide-energy-gap host layer may not be the best option. The reason is that the polarity of a single host could be not suitable for both blue TADF sensitizer and blue MR-TADF emitter at the same time. Generally, the weak D‒A interaction of blue TADF sensitizers in low-polar hosts will have a negative impact on the TADF property and the RISC process, and the increasing triplet-involved quenching process in blue TADF sensitizers will decrease EL efficiencies. Therefore, most blue TADF sensitizers prefer high-polar hosts to improve the TADF property. However, the high-polar hosts will lead to an enhanced CT effect of the blue MR-TADF emitters, which will broaden the EL spectra and decrease the PLQYs of the blue MR-TADF emitters. Therefore, blue MR-TADF emitters require low-polar hosts to prevent the broadened EL spectra and decreased PLQY.

**Fig. 1:**
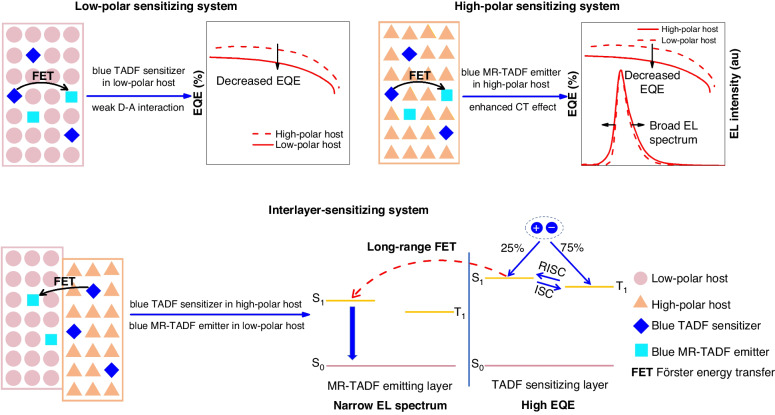
Schematic illustrations of different sensitizing systems for blue MR-TADF emitters. Schematic illustrations of low-polar sensitizing system, high-polar sensitizing system, and interlayer-sensitizing system for blue MR-TADF emitters

In this work, we wish to report an effective interlayer-sensitizing configuration for blue MR-TADF emitters, in which TADF sensitizer and MR-TADF emitter are separated into two adjacent emitting layers (EMLs) with different hosts, and MR-TADF emitters can be sensitized by TADF sensitizers via long-range Förster energy transfer (FET), as displayed in Fig. 1. Based on this interlayer sensitization strategy, high-performance hyperfluorescence OLEDs using different blue MR-TADF emitters are prepared, furnishing strong blue light with narrow EL spectra and outstanding external quantum efficiencies (EQEs) of up to 38.8%, which are apparently improved in comparison with those of unsensitized devices. These results demonstrate that this interlayer sensitization strategy is applicable for the fabrication of blue hyperfluorescence OLEDs with high EL efficiency and high color purity simultaneously.

## Results

**Fig. 2:**
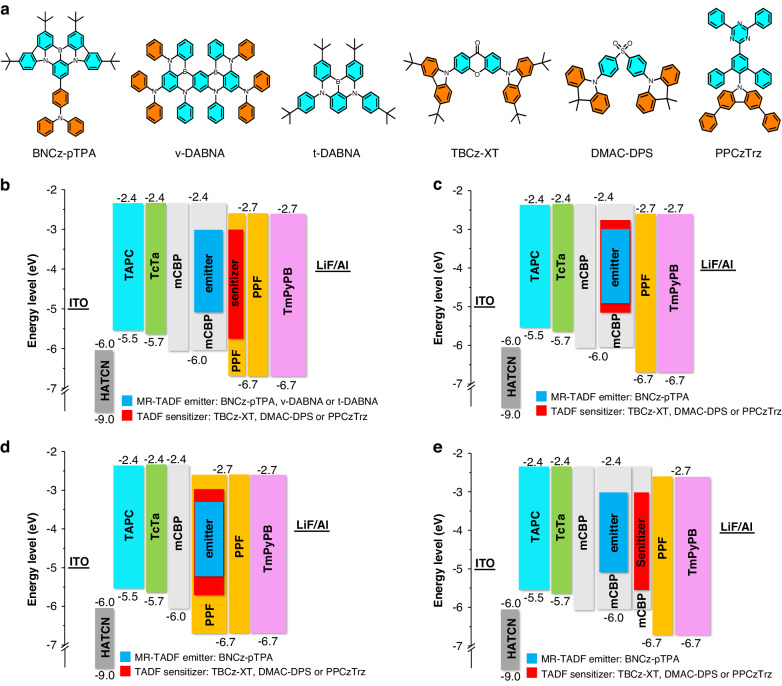
**Materials information and device configurations of OLEDs.** **a** Molecular structures of MR-TADF emitters and TADF sensitizers employed in this work. Device configurations of **b** devices IS1‒IS9, **c** devices S1‒S3, **d** devices S4‒S6, and **e** devices S7‒S9

To validate the feasibility of this interlayer sensitization strategy, efficient energy transfer from TADF sensitizers to MR-TADF emitters should be ensured^30^. According to the influencing factors in the FET radius (*R*_0_) formula given in supplementary information, the *Φ*_PL_s of the TADF sensitizers and the spectral overlaps between the PL spectra of the TADF sensitizers and the absorption spectra of the MR-TADF emitters should be considered to realize efficient FET. Although the key requirements might vary depending on different sensitizing systems, these two requirements are both taken into consideration for the construction of the sensitizing systems in this work. Initially, as a proof of concept, a robust blue MR-TADF molecule BNCz-pTPA^31^ with high EL performance is chosen as the emitter, and three efficient blue TADF materials (TBCz-XT, DMAC-DPS, and PPCzTrz)^32–34^ with totally different molecular structures are selected as the sensitizers. The molecular structures of these materials are displayed in Fig. 2a. As displayed in Table S2 and Fig. S1, three chosen blue TADF sensitizers all have *Φ*_PL_s of over 90%, and the PL spectra of these TADF sensitizers have large overlaps with the absorption spectra of the MR-TADF emitters.

The hyperfluorescence devices (IS1, IS2, and IS3) are fabricated with a configuration of ITO/HATCN (5 nm)/TAPC (50 nm)/TCTA (5 nm)/mCBP (5 nm)/1 wt% BNCz-pTPA: mCBP (10 nm)/sensitizing layer (2 nm)/PPF (5 nm)/TmPyPB (40 nm)/LiF (1 nm)/Al (120 nm) (Fig. 2b). Thereinto, the sensitizing layers are configured as 20 wt% TBCz-XT: PPF, 20 wt% DMAC-DPS: PPF and 10 wt% PPCzTrz: PPF for devices IS1, IS2 and IS3, respectively, in which indium-tin oxide (ITO) is the transparent anode, 1,4,5,8,9,11-hexaazatriphenylenehexacabonitrile (HATCN) is the hole injection layer, 4,4’-cyclohexylidenebis[*N*,*N*-bis(4-methylphenyl)aniline] (TAPC) is the hole-transporting layer, tris[4-(carbazol-9-yl)phenyl]amine (TCTA) works as the hole-transporting layer as well as the electron-blocking layer, 3,3-di(9H-carbazol-9yl)biphenyl (mCBP) is used as the host for MR-TADF emitter BNCz-pTPA as well as the electron-blocking layer, bis-(diphenylphosphoryl)-dibenzo[b,d]-furan-synonym (PPF) is used as the host for TADF sensitizers (TBCz-XT, DMAC-DPS and PPCzTrz) as well as the hole-blocking layer, 1,3,5-tri(m-pyrid-3-yl-phenyl)benzene (TmPyPB) is the electron-transporting layer, and LiF/Al serves as cathode. The thickness of the sensitizing layer should be kept within the *R*_0_ of the blue TADF sensitizers, commonly in the range of 2‒4 nm^35,36^, otherwise parts of energy cannot be transferred from TADF sensitizers to MR-TADF emitters, leading to the appearance of intrinsic emissions of TADF sensitizers. Moreover, to prevent concentration quenching, the doping concentration of MR-TADF emitter is usually controlled at a very low level, which will also cause incomplete energy transfer of the TADF sensitizers. Consequently, in order to cover more MR-TADF molecules within the energy transfer range of the TADF sensitizers, the sensitizing layer should be designed as thin as possible. However, sufficient electrogenerated excitons are essential for high EL performances, so the thickness of the sensitizing layer cannot be too small. Here, considering the *R*_0_ of these blue TADF sensitizers (Table S2) and the potential incomplete energy transfer caused by doping concentration difference, the thickness of the sensitizing layer is set as 2 nm in these devices.

**Fig. 3:**
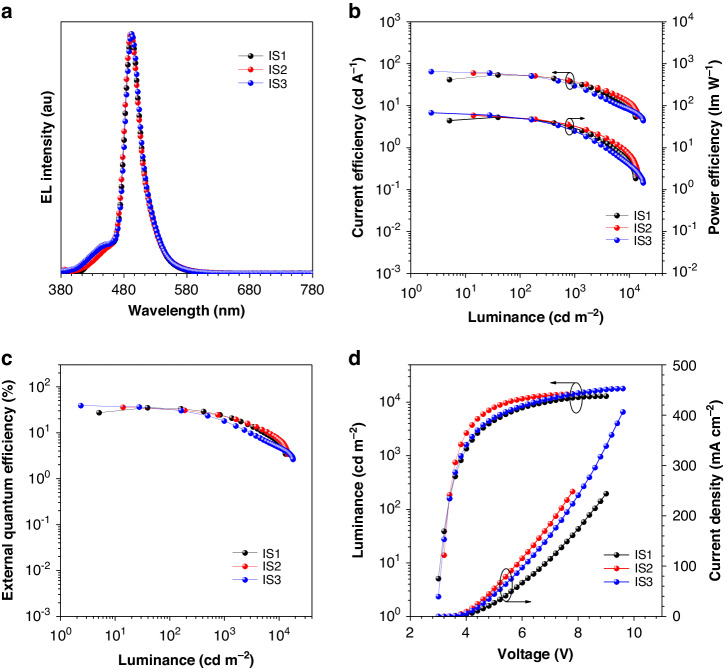
**Device data and EL performance of devices IS1‒IS3.** **a** EL spectra at 5 V, **b** current efficiency and power efficiency versus luminance curves, **c** external quantum efficiency versus luminance curves, and **d** luminance and current density versus voltage curves of devices IS1‒IS3

As shown in Fig. 3b‒d and Table 1, devices IS1, IS2, and IS3 possess low turn-on voltages of 3.0‒3.2 V, implying small carrier injection barriers and efficient recombination of holes and electrons in the devices. Devices IS1, IS2, and IS3 attain outstanding peak EQEs of 35.0%, 35.7%, and 38.8%, obviously higher than that of the unsensitized device (Fig. S2, Table S1). Such high EQEs are attributed not only to the high PLQY (95%) and large horizontal dipole ratio (87%) of the MR-TADF emitter BNCz-pTPA, but also to the efficient energy transfer from TADF sensitizer to MR-TADF emitter. The EQEs of devices IS1, IS2, and IS3 are still kept at 33.8%, 32.0%, and 31.7% at 100 cd m^‒2^ luminance, and 23.2%, 22.6%, and 17.9% at 1000 cd m^‒2^ luminance. The EL spectra of devices IS1, IS2, and IS3 at 5 V are presented in Fig. 3a. It can be seen that the three devices all exhibit sky-blue EL emissions with peaks at 492 nm and narrow FWHMs of 30 nm, demonstrating high color purity.

**Table 1:** EL performances of hyperfluorescence OLEDs

Device	λ_EL_ [nm]	*V*_on_ [V]	*L*_max_ [cd m^−2^]	CE [cd A^−1^]	PE [lm W^−1^]	EQE [%]	CIE (*x*, *y*)	FWHM [nm]
						Max/100 cd m^‒2^/1000 cd m^‒2^		
IS1	492	3.0	13080	54.3	53.3	35.0/33.8/23.2	(0.109, 0.273)	30
IS2	492	3.2	14290	60.1	59.0	35.7/32.0/22.6	(0.111, 0.314)	30
IS3	492	3.0	18020	65.2	68.2	38.8/31.7/17.9	(0.111, 0.314)	30
IS4	470	3.2	9362	20.9	19.8	24.0/20.3/13.0	(0.131, 0.104)	19
IS5	470	3.2	12680	26.9	24.8	29.6/25.1/18.0	(0.131, 0.113)	18
IS6	470	3.2	13030	29.1	28.6	28.6/18.6/11.8	(0.133, 0.128)	19
IS7	462	3.0	7588	22.8	23.9	25.0/13.1/6.4	(0.140, 0.104)	32
IS8	462	3.2	7813	26.3	25.8	26.2/12.6/6.0	(0.135, 0.130)	29
IS9	462	3.2	8001	26.9	26.4	25.9/12.1/5.4	(0.141, 0.125)	28
S1	492	3.0	24740	52.8	55.3	29.3/24.9/16.8	(0.100, 0.367)	29
S2	492	3.0	24720	52.7	51.7	29.7/29.3/22.1	(0.098, 0.363)	29
S3	494	3.4	27930	63.1	52.3	32.9/32.1/22.6	(0.097, 0.410)	29
S4	492	3.0	28250	52.6	54.4	29.5/28.0/19.6	(0.099, 0.359)	32
S5	494	3.0	20290	59.3	62.0	31.8/30.7/23.1	(0.101, 0.386)	32
S6	494	3.2	19610	66.6	65.4	33.2/21.5/10.9	(0.105, 0.421)	32
S7	490	3.0	18160	30.6	30.0	23.4/21.9/14.6	(0.123, 0.199)	31
S8	490	3.2	14090	38.2	35.3	23.8/22.2/14.8	(0.131, 0.284)	32
S9	490	3.2	19990	35.0	34.3	25.8/21.5/12.8	(0.124, 0.219)	31

*V*_on_ = turn-on voltage at 1 cd m^−2^; *L*_max_ = maximum luminance; *CE/PE/EQE* current efficiency/power efficiency/external quantum efficiency; *λ*_EL_ = EL peak; *CIE* Commission Internationale de l’Eclairage coordinates; *FWHM* full-width at half-maximum

**Fig. 4:**
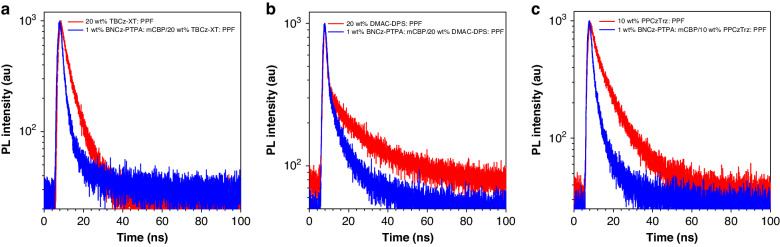
**Mechanism study of the sensitizing process.** Transient PL decay curves of **a** doped films I and IV, **b** doped films II and V, and **c** doped films III and VI, detected at 450 nm

To further explore the working mechanism inside the interlayer-sensitized hyperfluorescence devices, transient PL decay curves of the doped film I of 20 wt% TBCz-XT: PPF (20 nm), doped film II of 20 wt% DMAC-DPS: PPF (20 nm), doped film III of 10 wt% PPCzTrz: PPF (20 nm), doped film IV of 1 wt% BNCz-pTPA: mCBP (10 nm)/20 wt% TBCz-XT: PPF (2 nm), doped film V of 1 wt% BNCz-pTPA: mCBP (10 nm)/20 wt% DMAC-DPS: PPF (2 nm), and doped film VI of 1 wt% BNCz-pTPA: mCBP (10 nm)/10 wt% PPCzTrz: PPF (2 nm) are collected at 450 nm. The transient PL decay curves of the six films are displayed in Fig. 4, and the PL spectra of these films (tested using the transient PL excitation wavelength 340 nm) are provided in Fig. S3. The prompt lifetimes of TADF sensitizers in films IV, V, and VI are tested to be 5.9, 8.8, and 6.5 ns, apparently shorter than the corresponding values of 8.2, 17.0, and 10.3 ns in films I, II, and III, respectively. The shorter prompt lifetime is attributed to the presence of the MR-TADF molecules, which provides an additional singlet relaxation path for the TADF sensitizers, indicative of the FET process from TADF sensitizers to BNCz-pTPA emitter^37^.

Subsequently, for comparison, the co-phase sensitized hyperfluorescence devices (S1‒S3) are designed by co-doping MR-TADF emitters and TADF sensitizers into a single wide-energy-gap host. Firstly, the devices using low-polar wide-energy-gap host mCBP are fabricated with a configuration of ITO/HATCN (5 nm)/TAPC (50 nm)/TCTA (5 nm)/mCBP (5 nm)/EML (20 nm)/PPF (5 nm)/TmPyPB (40 nm)/LiF (1 nm)/Al (120 nm) (Fig. 2c). Thereinto, the EMLs are 1 wt% BNCz-pTPA: 20 wt% TBCz-XT: mCBP, 1 wt% BNCz-pTPA: 20 wt% DMAC-DPS: mCBP and 1 wt% BNCz-pTPA: 10 wt% PPCzTrz: mCBP for devices S1, S2 and S3, respectively. The peak EQEs of devices S1, S2, and S3 are 29.3%, 29.7%, and 32.9% (Fig. S4, Table 1), obviously lower than those of the interlayer-sensitized devices, and some EQEs are even lower than those of the unsensitized devices. These results suggest the co-phase sensitizing configuration of co-doping TADF sensitizers and MR-TADF emitters in a low-polar host is not as efficient as the interlayer-sensitizing configuration, probably attributed to the low-polar host is not suitable for the TADF sensitizers. Blue TADF materials usually have weak D‒A interaction and large Δ*E*_ST_s in low-polar hosts, which are unfavorable for achieving a fast RISC process and efficient TADF characteristic, and the increasing triplet-involved quenching process in blue TADF sensitizers will decrease EL efficiencies. However, high-polar hosts are conducive to lowering Δ*E*_ST_s to improve their TADF property^38^.

To confirm this, control devices (SB1‒SB6) with TADF sensitizers doped in low-polar host mCBP and high-polar host PPF, respectively, are constructed. Devices SB1‒SB6 have the same structures as the co-phase sensitized hyperfluorescence devices except for the EMLs that are designed as 20 wt% TBCz-XT: mCBP, 20 wt% TBCz-XT: PPF, 20 wt% DMAC-DPS: mCBP, 20 wt% DMAC-DPS: PPF, 10 wt% PPCzTrz: mCBP and 10 wt% PPCzTrz: PPF, respectively. Devices with three TADF sensitizers doped in low-polar host mCBP show peak EQEs of 17.8%, 19.9% and 20.2%, much lower than those in high-polar host PPF (30.1%, 29.8% and 34.1%) (Fig. S5‒S7, Table S1), accounting for the lower EL efficiencies of the sensitized hyperfluorescence devices with low-polar mCBP host.

Since the blue TADF sensitizers prefer high-polar hosts to low-polar ones, the co-phase sensitized hyperfluorescence devices (S4‒S6) with the same configuration as devices S1‒S3 except for using the high-polar host PPF are also prepared (Fig. 2d). Devices S4‒S6 show peak EQEs of 29.5%, 31.8% and 33.2% (Fig. S8, Table 1), respectively, slightly higher than those of devices S1‒S3, confirming the high-polar PPF host indeed has positive effect on improving EL efficiencies. However, these EQEs are still obviously lower than those of the interlayer-sensitized hyperfluorescence devices IS1‒IS3. In addition, devices S4‒S6 exhibit EL spectra with FWHMs of 32 nm, broader than those of the unsensitized devices (28 nm) as well as the interlayer-sensitized devices (30 nm). The lower EL efficiencies and broader EL spectra of devices S4‒S6 should be attributed to the decreased PLQY and strengthened CT effect of the MR-TADF emitter caused by the high-polar host PPF.

To confirm the discussions above, the PLQY and PL spectra of the doped films of 1 wt% BNCz-pTPA: PPF and 1 wt% BNCz-pTPA: mCBP are measured. The PLQY of the film of 1 wt% BNCz-pTPA: PPF is 85%, lower than that of the film of 1 wt% BNCz-pTPA: mCBP (95%), which is responsible for the lower EQEs of the devices with high-polar PPF host. Meanwhile, as shown in Fig. S9, the PL spectrum of the film of 1 wt% BNCz-pTPA: PPF has a FWHM of 29 nm, broader than that of the film of 1 wt% BNCz-pTPA: mCBP (26 nm), evidencing that high-polar host will broaden the EL spectrum of the MR-TADF emitter.

It has been reported that a similar device configuration with two separated adjacent phases could reduce short-range Dexter energy transfer (DET), which is beneficial for higher EL efficiencies^39^. Differently, the interlayer-sensitizing device configuration in this work is specifically proposed for optimizing blue MR-TADF emitters and the main reason for the improved device efficiencies is that the blue MR-TADF emitter and blue TADF sensitizer are both doped in wide-energy-gap hosts with suitable polarity. To exclude the influence of DET, the interlayer-sensitized hyperfluorescence devices (S7‒S9) using the same host mCBP are fabricated for comparison. The structures of devices S7‒S9 are the same as those of devices IS1‒IS3 except that the sensitizing layers are configured as 20 wt% TBCz-XT: mCBP, 20 wt% DMAC-DPS: mCBP and 10 wt% PPCzTrz: mCBP for devices S7, S8 and S9 (Fig. 2e), respectively. The peak EQEs of devices S7, S8 and S9 are 23.4%, 23.8% and 25.8% (Fig. S10, Table 1), much lower than those of interlayer-sensitized hyperfluorescence devices IS1‒IS3, and even lower than those of co-phase sensitized hyperfluorescence devices S1-S3. These results suggest that the efficiency improvement is attributed to the suitable polarity of the wide-energy-gap hosts instead of the decrease of DET. Besides, it can be seen from Fig. S10a that devices S7‒S9 all show obvious emissions from TADF sensitizers. The emissions of TADF sensitizers (TBCz-XT and PPCzTrz) in devices S7 and S9 are peaked at about 450 nm, corresponding to the EL peaks of devices SB1 and SB5. The EL peak of the TADF sensitizer DMAC-DPS in mCBP host (device SB3) is located at about 470 nm, close to the EL peak of MR-TADF emitter BNCz-pTPA (about 490 nm). So, the emission spectrum of TADF sensitizer DMAC-DPS is mostly covered by the emission spectrum of the MR-TADF emitter, which makes the right half of the EL spectrum of device S8 obviously broader than those of devices S7 and S9.

**Fig. 5:**
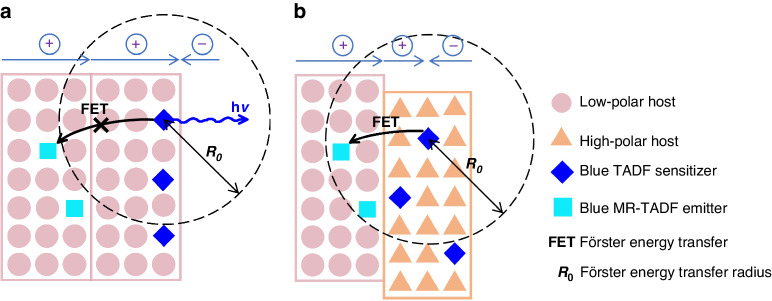
**The exciton behaviors of the interlayer-sensitizing systems.** **a** The same hosts for TADF sensitizers and MR-TADF emitters. **b** The different hosts for TADF sensitizers and MR-TADF emitters

To explain the obvious emissions of TADF sensitizers generated in devices S7‒S9, the carrier mobility of different doped layers in devices S7‒S9 (1 wt% BNCz-pTPA: mCBP, 20 wt% TBCz-XT: mCBP, 20 wt% DMAC-DPS: mCBP and 10 wt% PPCzTrz: mCBP) are investigated via space charge limited current (SCLC) method^40^. Hole-only devices with the configuration of ITO/MoO_3_ (10 nm)/doping layer (80 nm)/MoO_3_ (10 nm)/Al (120 nm) and electron-only devices with the configuration of ITO/TmPyPB (10 nm)/doping layer (80 nm)/TmPyPB (10 nm)/LiF (1 nm)/Al (120 nm), respectively, are fabricated, in which MoO_3_ and TmPyPB functioned as buffer layers to shield off electrons and holes between the doped layers and the electrodes, respectively. Owing to the much thicker doped layers (80 nm), the influence of the buffer layers can be excluded. The electric field-dependent mobilities of the different doped layers are displayed in Figs. S11, S12. It can be seen that all the hole mobilities (10^−3^‒10^−2^ cm^2^ V^−1^ s^−1^) in devices S7‒S9 are several orders of magnitude larger than the electron mobilities (10^−6^‒10^−5^ cm^2^ V^−1^ s^−1^). In consequence, most excitons will recombine at the interface between the sensitizing layer and the hole-blocking layer, as shown in Fig. 5a. The thickness of the sensitizing layer is set as 2 nm, so the energy of TADF sensitizers can only be transferred to MR-TADF molecules within the thickness range of (*R*_0_‒2) nm through FET, and considering the low doping concentration of MR-TADF emitter, the excitons generated on the TADF sensitizers will be more than the MR-TADF molecules that can accept energy. Therefore, parts of the excitons cannot be transferred to MR-TADF molecules and thus will undergo direct radiative decay on TADF sensitizers. For comparison, the carrier mobilities of the different sensitizing layers in devices IS1‒IS3 (20 wt% TBCz-XT: PPF, 20 wt% DMAC-DPS: PPF and 10 wt% PPCzTrz: PPF) are also investigated and displayed in Fig. S13. Different from devices S7‒S9, the hole mobilities (10^−5^‒10^−4^ cm^2^ V^−1^ s^−1^) of the sensitizing layers in devices IS1‒IS3 are close to their electron mobilities (10^−6^‒10^−4^ cm^2^ V^−1^ s^−1^). Therefore, after holes quickly passing through the EML (1 wt% BNCz-pTPA: mCBP) with high hole mobility (10^−3^‒10^−2^ cm^2^ V^−1^ s^−1^), the holes and electrons will move with similar mobilities in the sensitizing layer and recombine into excitons, and at this time the thickness range for the MR-TADF molecules to accept energy is larger than (*R*_0_‒2) nm, as displayed in Fig. 5b. Consequently, compared with devices S7-S9, the emissions of TADF sensitizers in devices IS1-IS3 are greatly reduced, which further proves the importance of the appropriate host combination for TADF sensitizers and MR-TADF emitters in the interlayer-sensitized hyperfluorescence devices.

According to the previous reports, using exciplex hosts in the sensitizing system is an effective approach to optimize the EL efficiencies of the MR-TADF emitters, so the co-phase sensitized hyperfluorescence devices (S10‒S12) using corresponding exciplex host (mCBP: PPF mixture) are constructed for comparison. Devices S10‒S12 have the same structures as the co-phase sensitized hyperfluorescence devices S1‒S6 except for the EMLs that are designed as 1 wt% BNCz-pTPA: 20 wt% TBCz-XT: 39.5 wt% mCBP: 39.5 wt% PPF, 1 wt% BNCz-pTPA: 20 wt% DMAC-DPS: 39.5 wt% mCBP: 39.5 wt% PPF and 1 wt% BNCz-pTPA: 10 wt% PPCzTrz: 44.5 wt% mCBP: 44.5 wt% PPF, respectively. As shown in Table S3 and Fig. S14, the peak EQEs of devices S10‒S12 are 32.0%, 30.8%, and 33.7%, respectively, which are obviously inferior to those of the interlayer-sensitized hyperfluorescence devices IS1‒IS3 (35.0‒38.8%), indicating the superiority of interlayer-sensitized hyperfluorescence devices in optimizing the EL efficiencies of blue MR-TADF emitters.

The above results and analyses manifest that the interlayer-sensitizing system is advantageous to optimize some blue MR-TADF emitters compared with the co-phase sensitizing system using a single wide-energy-gap host. Then, the universality of this strategy is examined by introducing it to other blue MR-TADF emitters of *v*-DABNA and *t*-DABNA^41,42^, and above three TADF materials are still employed as sensitizers. The molecular structures of *v*-DABNA and *t*-DABNA are displayed in Fig. 2a, and the absorption spectra of *v*-DABNA and *t*-DABNA can well-overlapped with the emission spectra of these three TADF sensitizers (Fig. S1).

**Fig. 6:**
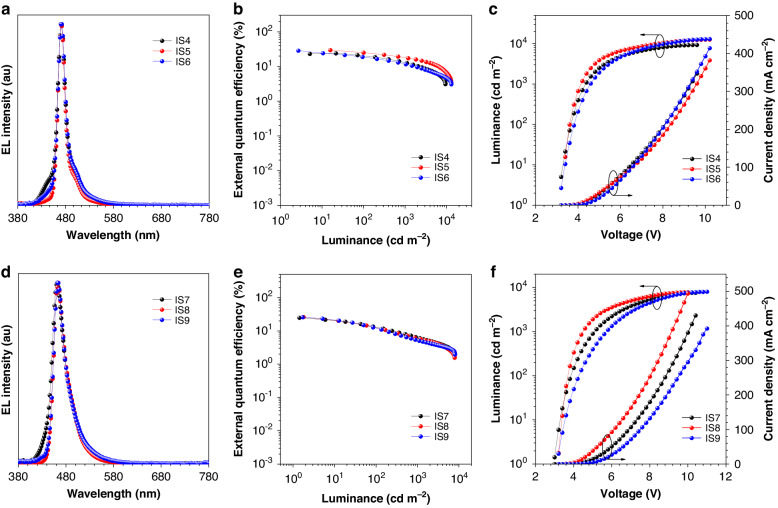
**Device data and EL performance of devices IS4-IS9.** **a** EL spectra at 5 V, **b** external quantum efficiency versus luminance curves, and **c** luminance and current density versus voltage curves of devices IS4‒IS6. **d** EL spectra at 5 V, **e** external quantum efficiency versus luminance curves, and **f** luminance and current density versus voltage curves of devices IS7‒IS9

The interlayer-sensitized hyperfluorescence devices (IS4‒IS6) using *v*-DABNA as emitter is ITO/HATCN (5 nm)/TAPC (50 nm)/TCTA (5 nm)/mCBP (5 nm)/1 wt% *v*-DABNA: mCBP (10 nm)/sensitizing layer (2 nm)/PPF (5 nm)/TmPyPB (40 nm)/LiF (1 nm)/Al (120 nm). The sensitizing layers are constructed as 20 wt% TBCz-XT: PPF, 20 wt% DMAC-DPS: PPF, and 10 wt% PPCzTrz: PPF for devices IS4, IS5 and IS6, respectively. As shown in Fig. 6a‒c and Table 1, devices IS4, IS5, and IS6 all demonstrate impressive EL performances with low turn-on voltages of 3.2 V and peak EQEs of 24.0%, 29.6% and 28.6%, respectively, higher than that of the unsensitized device of *v*-DABNA (19.0%). The EQEs still remain 20.3%, 25.1%, and 18.6% at 100 cd m^‒2^ luminance, and 13.0%, 18.0%, and 11.8% at 1000 cd m^‒2^ luminance, indicating the improvement of efficiency stability. The EL spectra at 5 V of devices IS4, IS5, and IS6 show peaks at 470 nm and small FWHMs of 18‒19 nm, indicative of excellent color purity.

Then, interlayer-sensitized hyperfluorescence devices (IS7‒IS9) employing *t*-DABNA as the emitter are fabricated and investigated in the same manner as above. The device structure is ITO/HATCN (5 nm)/TAPC (50 nm)/TCTA (5 nm)/mCBP (5 nm)/1 wt% *t*-DABNA: mCBP (10 nm)/sensitizing layer (2 nm)/PPF (5 nm)/TmPyPB (40 nm)/LiF (1 nm)/Al (120 nm). The sensitizing layers are 20 wt% TBCz-XT: PPF, 20 wt% DMAC-DPS: PPF, and 10 wt% PPCzTrz: PPF for devices IS7, IS8 and IS9, respectively. Devices IS7, IS8, and IS9 show pure-blue light with EL peaks at 462 nm and FWHMs of 32, 29, and 28 nm, respectively (Fig. 6d‒f, Table 1). High peak EQEs of 25.0%, 26.2%, and 25.9% are obtained in devices IS7, IS8, and IS9, respectively, much better than that of the unsensitized device of *t*-DABNA (14.1%). These devices also enjoy enhanced efficiency stability, with smaller efficiency roll-offs than the unsensitized device. These findings further manifest that the proposed interlayer sensitization strategy is effective and promising to improve the EL performances of blue MR-TADF emitters and keep high color purity.

There are weak emissions from the TADF sensitizers observed for the spectra of the interlayer-sensitized hyperfluorescence devices. The primary reason is the distance gradient between the TADF sensitizer and the MR-TADF emitter induced by the interlayer configuration. As the distance between the exciton on TADF sensitizer and MR-TADF molecule increases, the FET efficiency will decrease. Given the *R*_0_s of the TADF sensitizers are relatively small, ranging from 3.3‒4.5 nm, parts of excitons on TADF sensitizers are unable to transfer energy because of the low FET efficiency, leading to the emission of the TADF sensitizers, especially for the excitons recombined in proximity to the interface of sensitizing layer and hole-blocking layer (Fig. S10a). To further monitor the spectral changes with increased voltages, the EL spectra of devices IS1‒IS9 at different voltages are tested. As shown in Fig. S15‒S17, the emission of the TADF sensitizers increases slightly by rising voltage, which may result from the increased exciton concentration on TADF sensitizers but low FET efficiency at high current density, and the elongated distance between the TADF sensitizer and the MR-TADF emitter induced by the degradation at the EML/sensitizing layer interface.

To verify the degradation at the EML/sensitizing layer interface, the device lifetimes of the comparative devices LT1‒LT5 using BNCz-pTPA as the MR-TADF emitter and PPCzTrz as the TADF sensitizer are tested. For a more accurate understanding of device lifetime, certain improvements are made to the original device configuration to improve the device lifetimes, the device configuration is MoO_3_ (6.5 nm)/Tris-PCz (20 nm)/mCBP (20 nm)/EML/SF3-TRZ (10 nm)/50 wt% SF3-TRZ: Liq (30 nm)/LiF (1 nm)/Al (120 nm). Thereinto, the EMLs are configured as 1 wt% BNCz-pTPA: mCBP (10 nm)/10 wt% PPCzTrz: PPF (2 nm), 1 wt% BNCz-pTPA: 10 wt% PPCzTrz: mCBP (20 nm), 1 wt% BNCz-pTPA: 10 wt% PPCzTrz: PPF (20 nm), 1 wt% BNCz-pTPA: mCBP (10 nm)/10 wt% PPCzTrz: mCBP (2 nm), 1 wt% BNCz-pTPA: 10 wt% PPCzTrz: 44.5 wt% mCBP: 44.5 wt% PPF for devices LT1, LT2, LT3, LT4 and LT5, respectively, in which the MoO_3_ is the hole injection layer, 9-phenyl-3,6-bis(9-phenyl-9H-carbazol-3-yl)-9H-carbazole (Tris-PCz) is the hole-transporting layer, mCBP is used as the low-polar host as well as the electron-blocking layer, PPF is used as the high-polar host, 2-(9,9′-spirobi[fluoren]-3-yl)-4,6-diphenyl-1,3,5-triazine (SF3-TRZ) is the hole-blocking layer, 50 wt% SF3-TRZ: Liq is the electron-transporting layer and LiF/Al serves as cathode. As shown in Fig. S18, the LT_90_ values of devices LT1‒LT5 at the initial luminance of 1000 cd m^‒2^ are 0.2, 4.3, 0.3, 2.4 and 1.1 h, respectively. Due to the instability of the high-polar host PPF, the device lifetimes of devices LT1, LT3, and LT5 using high-polar host PPF are much shorter than those of devices LT2 and LT4 only using low-polar host mCBP. However, by comparing the device lifetimes of the co-phase sensitized device LT2 and the interlayer-sensitized device LT4, both of which only use mCBP as host, it can be found that the device lifetime of the interlayer-sensitized device LT4 (2.4 h) is shorter than that of the co-phase sensitized device LT2 (4.3 h), demonstrating the degradation at the EML/sensitizing layer interface.

## Discussion

In summary, to improve the EL performances of MR-TADF emitters, hyperfluorescence OLEDs were proposed, which are generally designed by co-doping MR-TADF emitters with conventional TADF sensitizers into a single host layer. However, the different requirements for host polarity of MR-TADF emitters and TADF sensitizers often lead to difficulties in acquiring high EQEs and narrow EL spectra simultaneously, particularly for blue hyperfluorescence OLEDs. To address this issue, herein, an interlayer sensitization strategy is reported for blue MR-TADF emitters. The interlayer-sensitizing system is designed by separating MR-TADF emitters and TADF sensitizers into two adjacent host layers with high polarity and low polarity, respectively. Blue MR-TADF emitters can be sensitized via the FET mechanism to realize high EQEs and maintain narrow EL spectra in the low-polar hosts. Based on the interlayer sensitization strategy, the state-of-the-art blue hyperfluorescence OLEDs with high color quality and outstanding EQEs of up to 38.8% are realized. This strategy is also effective for different blue MR-TADF emitters and different blue TADF sensitizers, demonstrating the feasibility and universality in the construction of high-performance blue hyperfluorescence OLEDs.

## Materials and methods

### Materials and instruments

The compounds HATCN, TAPC, TCTA, mCBP, PPF, TmPyPB, DMAC-DPS, PPCzTrz, *v*-DABNA, and *t*-DABNA were purchased from commercial sources. BNCz-pTPA and TBCz-XT were synthesized according to previously reported methods. UV-vis absorption spectra were measured on a Shimadzu UV-2600 spectrophotometer. PL spectra were recorded on a Horiba Fluoromax-4 spectrofluorometer. PL quantum yields were measured using a Hamamatsu absolute PL quantum yield spectrometer C11347 Quantaurus_QY. Transient PL decay spectra were measured using Edinburgh Instruments FLS1000 spectrometer.

### Device fabrication and characterization

The glass substrates precoated with a 90-nm layer of ITO with a sheet resistance of 15~20 Ω per square were successively cleaned in ultrasonic bath of acetone, isopropanol, detergent, and deionized water, respectively, taking 10 minutes for each step. Then, the substrates were totally dried in a 70 °C oven. Before the fabrication processes, in order to improve the hole injection ability of ITO, the substrates were treated by O_2_ plasma for 10 minutes. The vacuum-deposited OLEDs were fabricated under a pressure of <5 × 10^‒4^ Pa in the Suzhou Fangsheng OMV-FS380 or OMV-FS450 vacuum deposition system. Organic materials, LiF and Al were deposited at rates of 1~2 A s^‒1^, 0.1 A s^‒1^, and 5 A s^‒1^, respectively. The effective emitting area of the devices was 9 mm^2^, determined by the overlap between anode and cathode. The luminance–voltage–current density and EQE were characterized with a dual-channel Keithley 2614B source meter and a PIN-25D silicon photodiode. The EL spectra were obtained via an Ocean Optics USB 2000+ spectrometer with a Keithley 2614B source meter and a PhotoResearch PR670 spectroradiometer with a Keithley 2400 Source Meter. All the characterizations were conducted at room temperature in ambient conditions without any encapsulation, as soon as the devices were fabricated.

### Supplementary information

Supplementary Information
