# Supplementary material for: Promising interlayer sensitization strategy for the construction of high-performance blue hyperfluorescence OLEDs
Source: Light Sci Appl. 2024 Jun 13;13:139. doi: 10.1038/s41377-024-01490-6 (PMC11176382; doi:10.1038/s41377-024-01490-6)
Supplement: Supplementary file 1 — Supplementary Information [file 41377_2024_1490_MOESM1_ESM.docx]

Supplementary Information for

**Promising Interlayer Sensitization Strategy for the Construction of High-Performance Blue Hyperfluorescence OLEDs**

Jianghui Wang^1^, Peng Zou^1^, Letian Chen^1^, Zhentao Bai^1^, Hao Liu^1^, Wen-Cheng Chen^2^, Yanping Huo^2^, Ben Zhong Tang^3^ and Zujin Zhao^1^*

**Correspondence: Zujin Zhao* [*(mszjzhao@scut.edu.cn)*](mailto:(mszjzhao@scut.edu.cn))

*^1^State Key Laboratory of Luminescent Materials and Devices, Guangdong Provincial Key Laboratory of Luminescence from Molecular Aggregates, South China University of Technology, Guangzhou 510640, China*

*^2^School of Chemical Engineering and Light Industry, Guangdong University of Technology, Guangzhou, 510006, China*

*^3^School of Science and Engineering, Shenzhen Institute of Aggregate Science and Technology, The Chinese University of Hong Kong, Shenzhen, Guangdong 518172, China*

**Contents**

Förster energy transfer radius calculation

Carrier mobility calculation

Fig. S1 The absorption spectra (solid lines) of MR-TADF emitters in toluene solution (10^‒5^ mol L^‒1^) and the PL spectra (dash lines) of TADF sensitizers in doped films.

Fig. S2 EL performance of the MR-TADF emitters in the unsensitized devices.

Fig. S3 PL spectra of doped films I‒VI tested using the transient PL excitation wavelength 340 nm.

Fig. S4 Device data and EL performance of devices S1‒S3.

Fig. S5 Device data and EL performance of devices SB1‒SB2.

Fig. S6 Device data and EL performance of devices SB3‒SB4.

Fig. S7 Device data and EL performance of devices SB5‒SB6.

Fig. S8 Device data and EL performance of devices S4‒S6.

Fig. S9 PL spectra of BNCz-pTPA doped in mCBP or PPF.

Fig. S10 Device data and EL performance of devices S7‒S9.

Fig. S11 Electric field-dependent carrier mobilities of emitting layer 1 wt% BNCz-pTPA: mCBP.

Fig. S12 Electric field-dependent carrier mobilities of sensitizing layers using mCBP host.

Fig. S13 Electric field-dependent carrier mobilities of sensitizing layers using PPF host.

Fig. S14 EL performance of devices S10‒S12.

Fig. S15 The EL spectra of devices IS1‒IS3 at different voltages.

Fig. S16 The EL spectra of devices IS4‒IS6 at different voltages.

Fig. S17 The EL spectra of devices IS7‒IS9 at different voltages.

Fig. S18 Operational lifetimes of devices LT1‒LT5.

Table S1. EL performances of MR-TADF emitters and TADF sensitizers.

Table S2. Parameters for Förster energy transfer radius calculation of different TADF sensitizers.

References

Table S3. EL performances of the hyperfluorescence devices using exciplex host.

**Förster energy transfer radius calculation**

Förster energy transfer radius (*R*_0_) are calculated by the following formula (1).^[1,2]^

$R_{0}^{6}=\frac{9000\left( \ln10 \right)\kappa^{2}\Phi_{\mathrm{PL}}}{128\pi^{5}N_{A}n^{4}}\int_{0}^{\infty} F_{h}\left( \lambda\right)\varepsilon_{g}\left( \lambda\right)\lambda^{4}d\lambda$ (1)

where *κ^2^* is orientation factor (*κ^2^* is typically assumed to be 2/3 for the random orientation system), *Φ*_PL_ is the quantum yield of the TADF sensitizer, *N*_A_ is Avogadro’s number, *n* is the refractive index of the medium, $\int_{0}^{\infty} F_{h}\left( \lambda\right)\varepsilon_{g}\left( \lambda\right)\lambda^{4}d\lambda$is the spectral overlap integral between the PL of the TADF sensitizer and the absorption of the MR-TADF emitter, λ is the wavelength.

**Carrier mobility calculation**

The SCLC property can be described via the Mott-Gurney equation (2), and the carrier mobility (*μ*) of organic semiconductors can be calculated according to the Poole-Frenkel formula (3), where the *ε*_0_ is the vacuum permittivity (8.85 × 10^‒14^ C V^‒1^ cm^‒1^), *ε*_r_ is the relative dielectric constant (assumed to be 3.0 for organic semiconductor), *E* is the electric field, *μ*_0_ is the zero-field mobility, and *γ* is the Poole-Frenkel factor and *L* is the thickness of doping layers. By fitting the *J–V* curves in SCLC region to Equation (2), the *µ*_0_ and *γ* values are obtained, thus generating the field-dependent electron mobility according to Equation (3).^[3]^

$J=\frac{9}{8}\varepsilon_{0}\varepsilon_{r}\mu\frac{E^{2}}{L}=\frac{9}{8}\varepsilon_{0}\varepsilon_{r}\frac{V^{2}}{L^{3}}\mu_{0}\exp\left( 0.891\gamma\sqrt{\frac{V}{L}} \right)$ (2)

$\mu=\mu_{0}\exp\left( \gamma\sqrt{E} \right)$ (3)


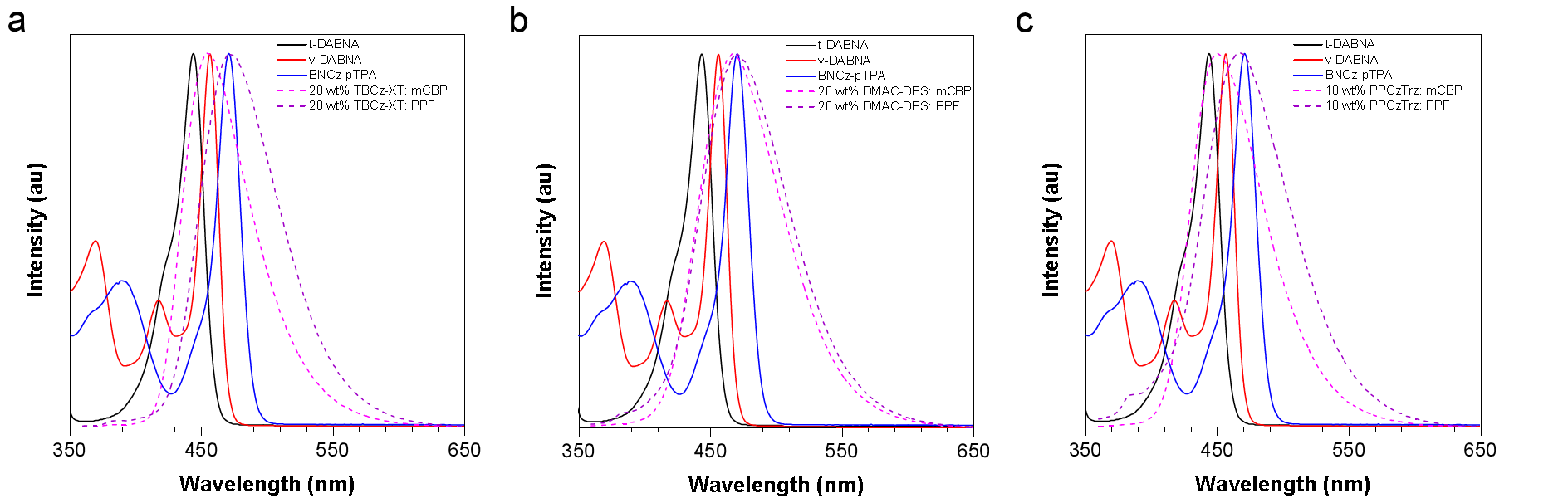


**Fig. S1 The absorption spectra (solid lines) of MR-TADF emitters in toluene solution (10^‒5^ mol L^‒1^) and the PL spectra (dash lines) of TADF sensitizers in doped films.** The absorption spectra of *t*-DABNA, *v*-DABNA and BNCz-pTPA and the PL spectra of **a** TBCz-XT, **b** DMAC-DPS and **c** PPCzTrz in doped films.


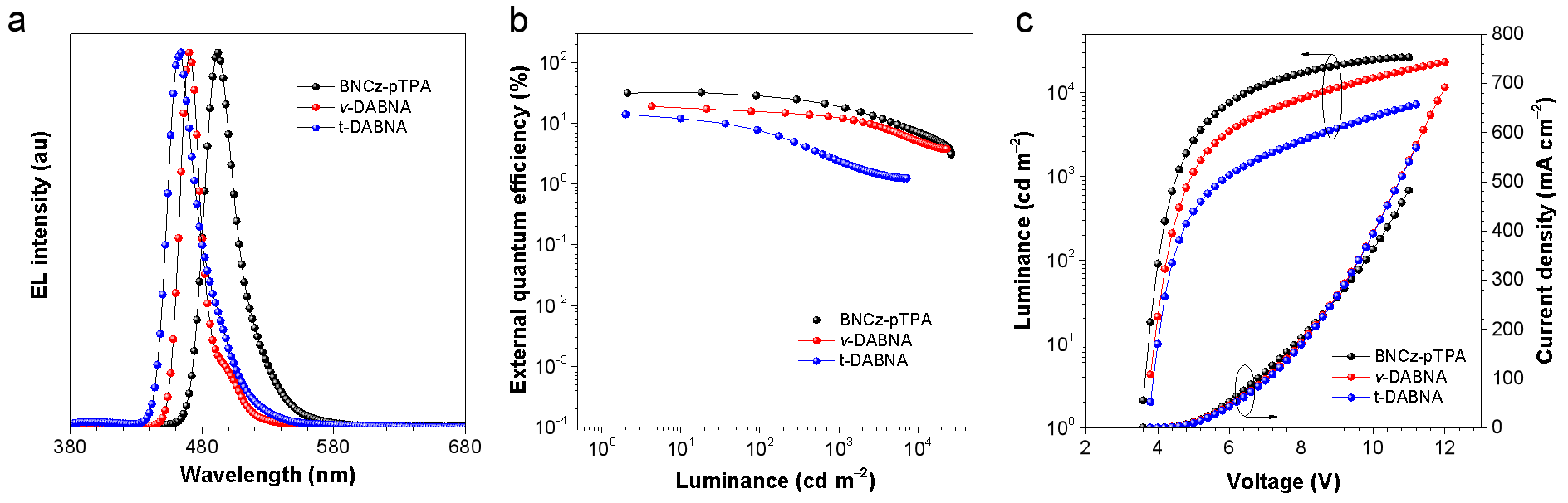


**Fig. S2** **EL performance of the MR-TADF emitters in the unsensitized devices.** **a** EL spectra at 5 V, **b** external quantum efficiency versus luminance curves, and **c** luminance and current density versus voltage curves.


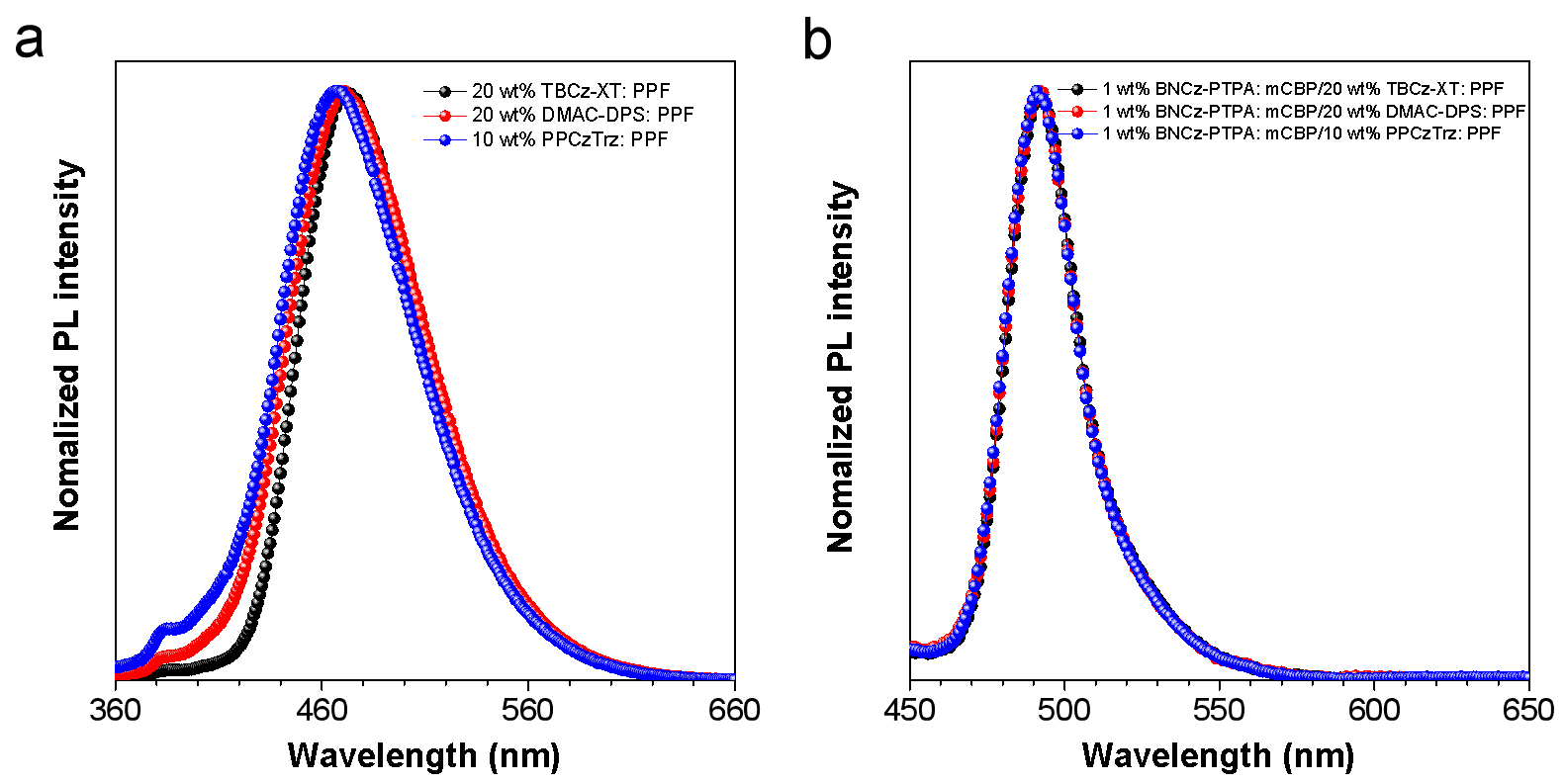


**Fig. S3 PL spectra of doped films I‒VI tested using the transient PL excitation wavelength 340 nm. a** PL spectra of doped films I‒III. **b** PL spectra of doped films IV‒VI.


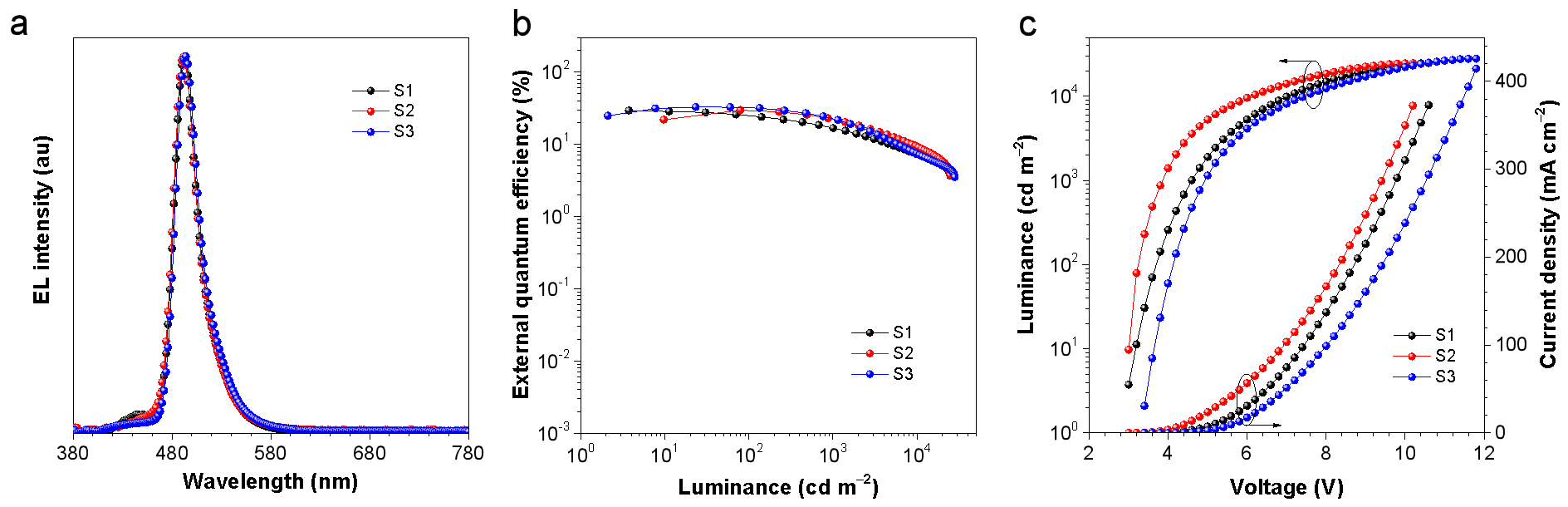


**Fig. S4** **EL performance of devices S1‒S3.** **a** EL spectra at 5 V, **b** external quantum efficiency versus luminance curves, and **c** luminance and current density versus voltage curves.

**
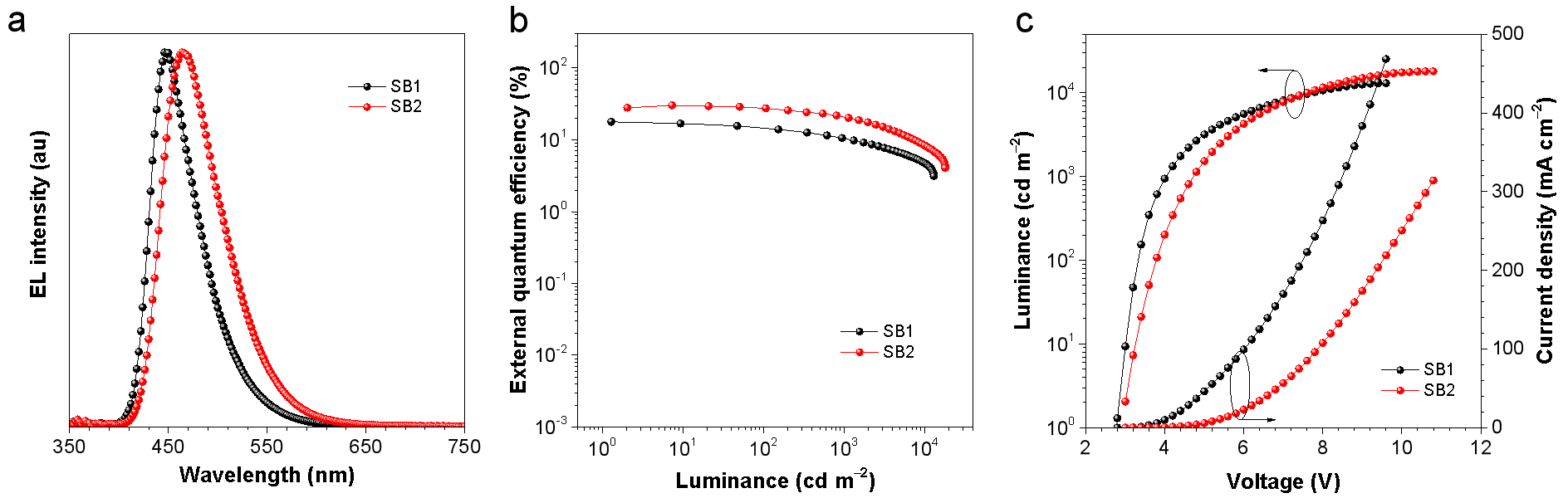
**

**Fig. S5** **EL performance of devices SB1‒SB2.** **a** EL spectra at 5 V, **b** external quantum efficiency versus luminance curves, and **c** luminance and current density versus voltage curves.


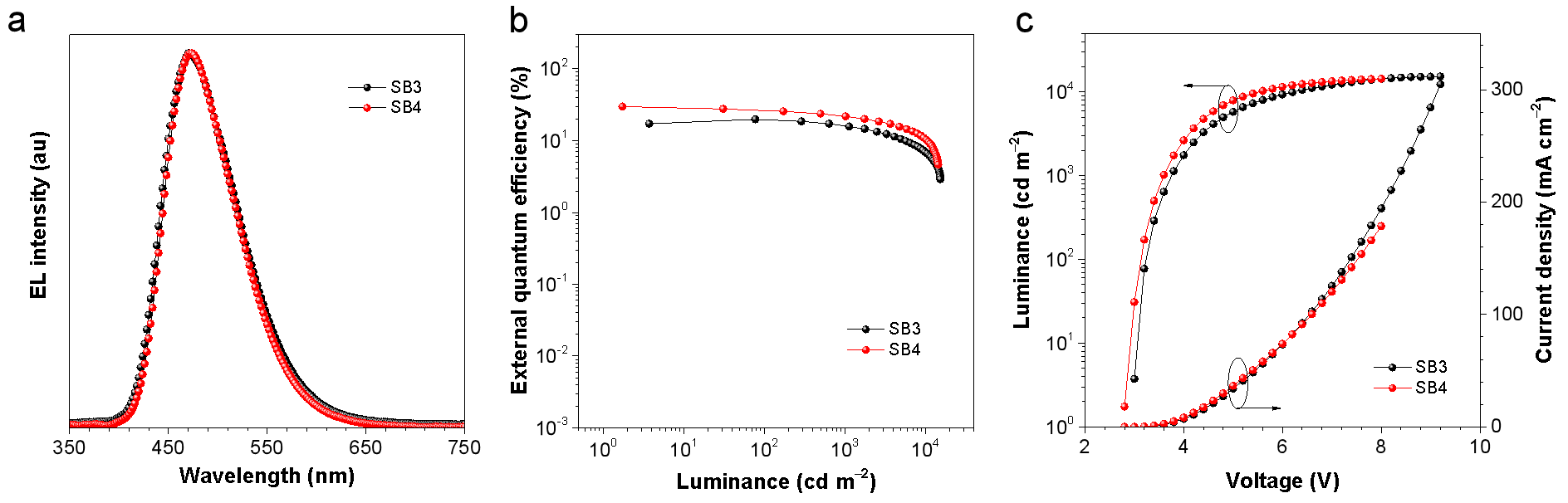


**Fig. S6** **EL performance of devices SB3‒SB4.** **a** EL spectra at 5 V, **b** external quantum efficiency versus luminance curves, and **c** luminance and current density versus voltage curves.


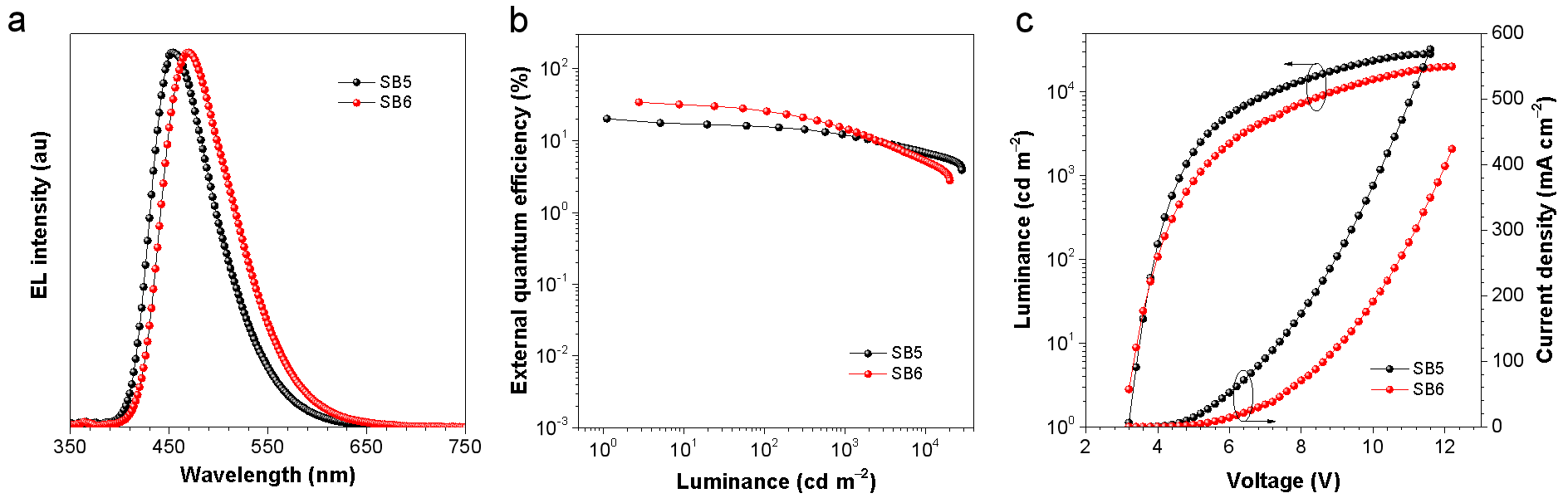


**Fig. S7** **EL performance of devices SB5‒SB6.** **a** EL spectra at 5 V, **b** external quantum efficiency versus luminance curves, and **c** luminance and current density versus voltage curves.


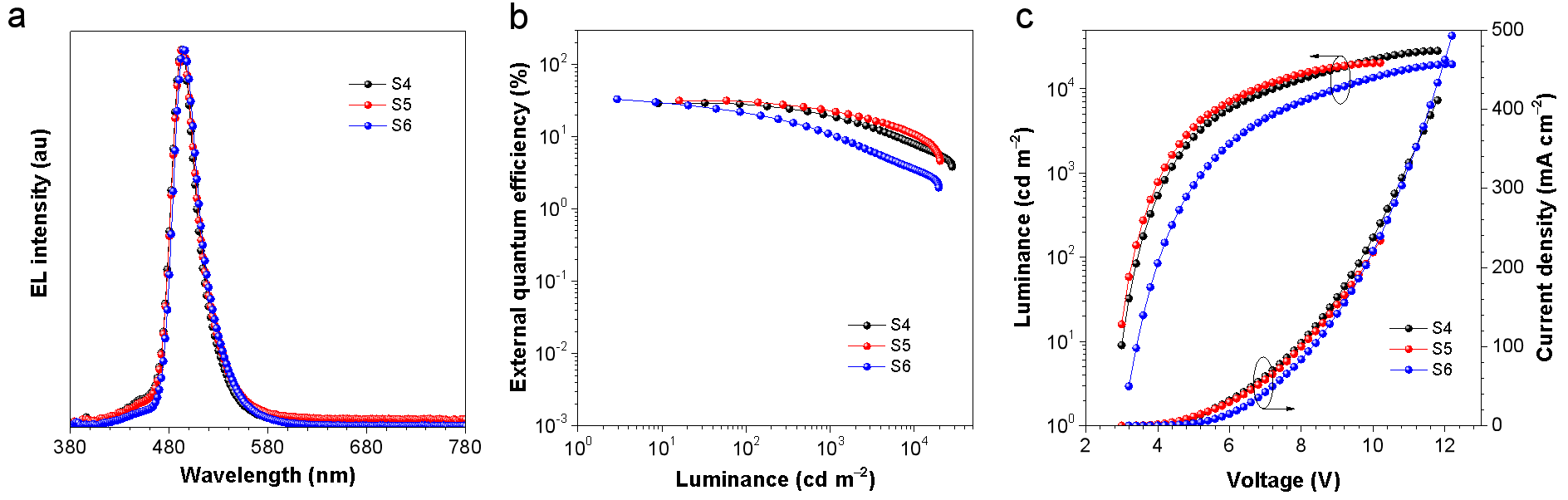


**Fig. S8** **EL performance of devices S4‒S6.** **a** EL spectra at 5 V, **b** external quantum efficiency versus luminance curves, and **c** luminance and current density versus voltage curves.





**Fig. S9 PL spectra of BNCz-pTPA doped in mCBP or PPF.**


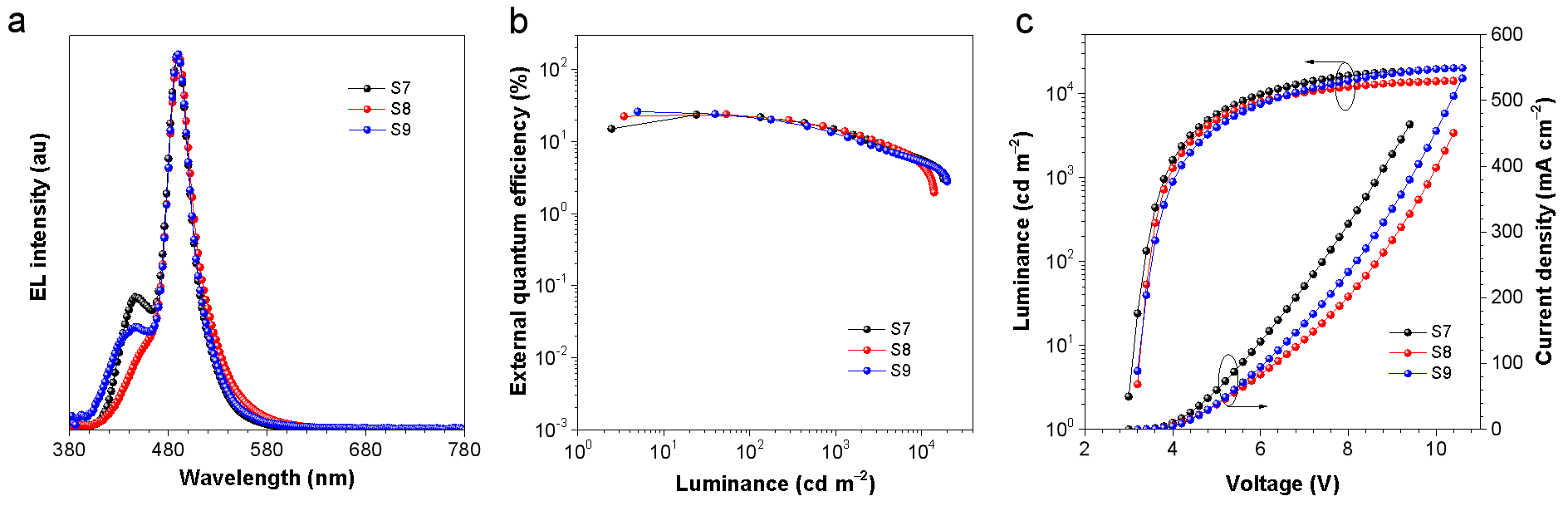


**Fig. S10** **EL performance of devices S7‒S9.** **a** EL spectra at 5 V, **b** external quantum efficiency versus luminance curves, and **c** luminance and current density versus voltage curves.


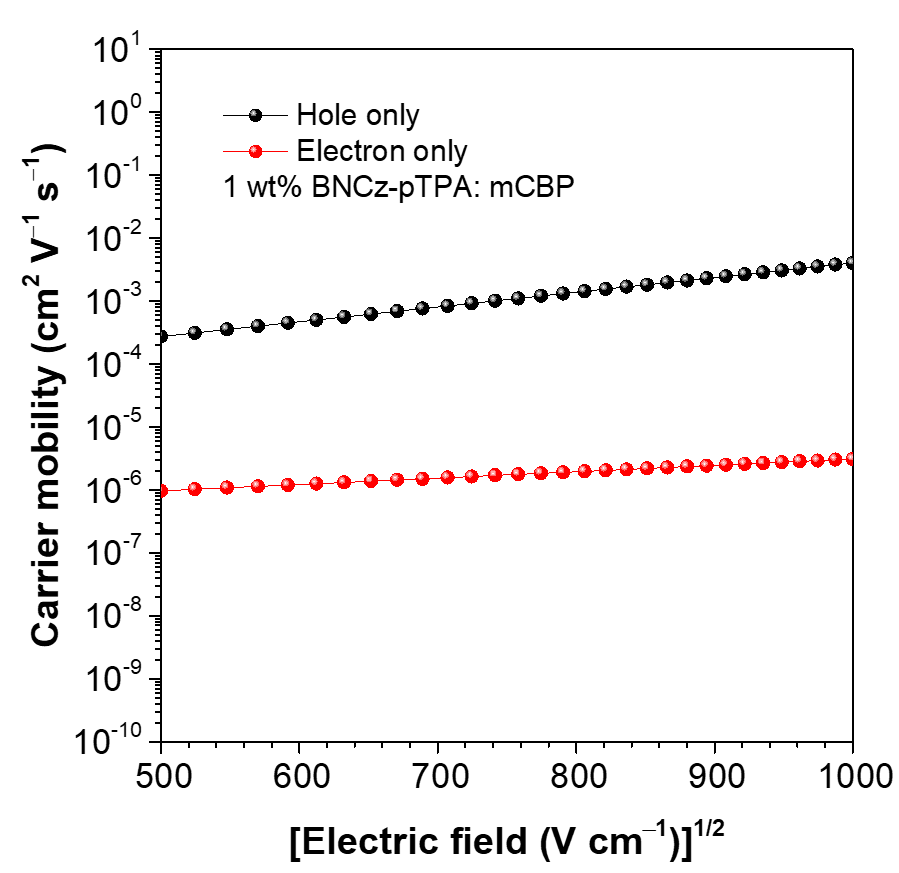


**Fig. S11 Electric field-dependent carrier mobilities of emitting layer 1 wt% BNCz-pTPA: mCBP.**


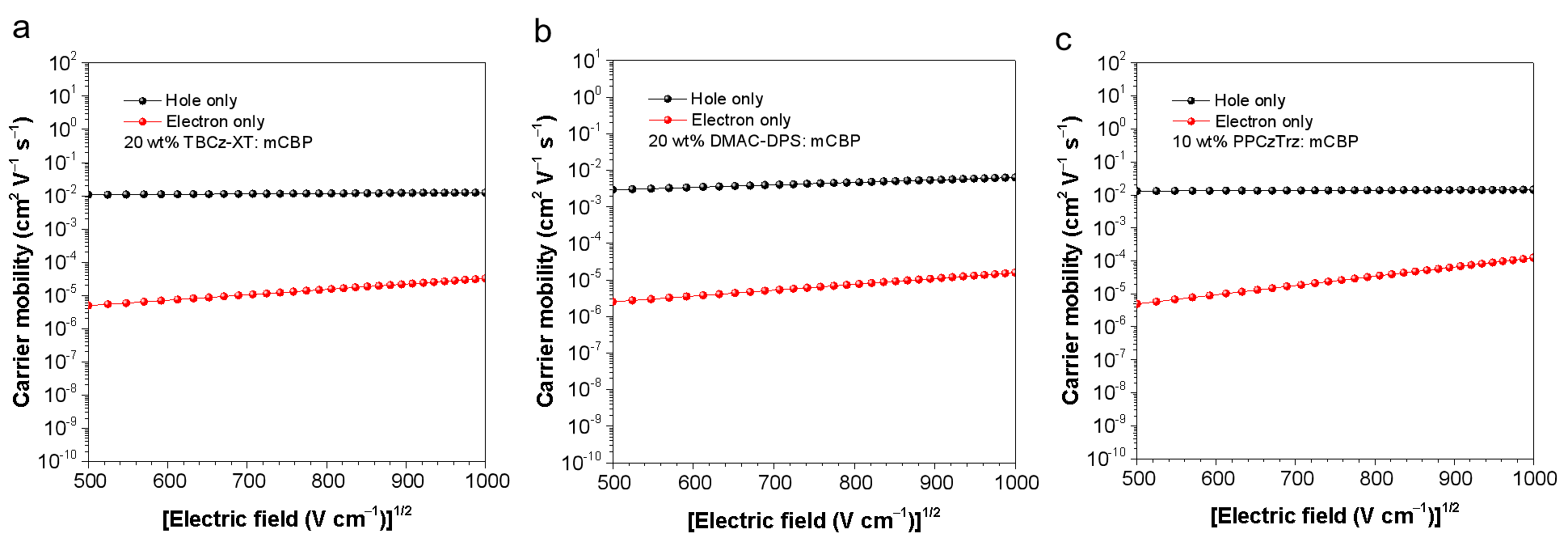


**Fig. S12 Electric field-dependent carrier mobilities of sensitizing layers using mCBP host.** Electric field-dependent carrier mobilities of **a** 20 wt% TBCz-XT: mCBP, **b** 20 wt% DMAC-DPS: mCBP, and **c** 10 wt% PPCzTrz: mCBP.


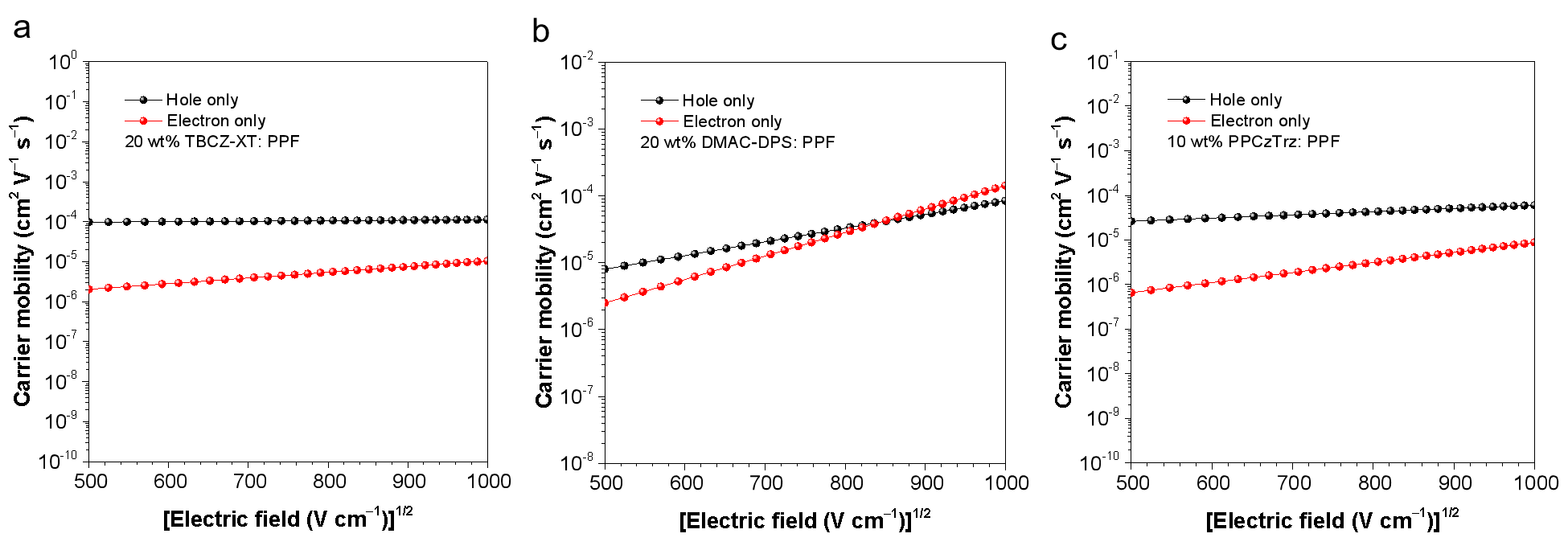


**Fig. S13 Electric field-dependent carrier mobilities of sensitizing layers using PPF host.** Electric field-dependent carrier mobilities of **a** 20 wt% TBCz-XT: PPF, **b** 20 wt% DMAC-DPS: PPF, and **c** 10 wt% PPCzTrz: PPF.


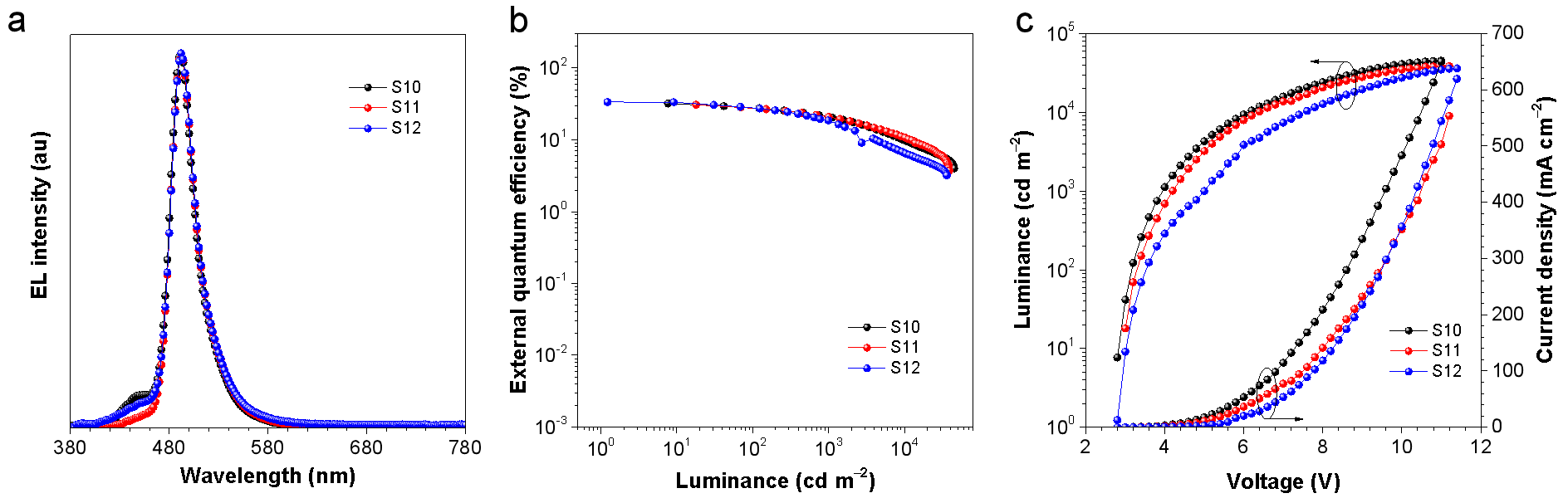


**Fig. S14** **EL performance of devices S10‒S12.** **a** EL spectra at 4 V, **b** external quantum efficiency versus luminance curves, and **c** luminance and current density versus voltage curves.


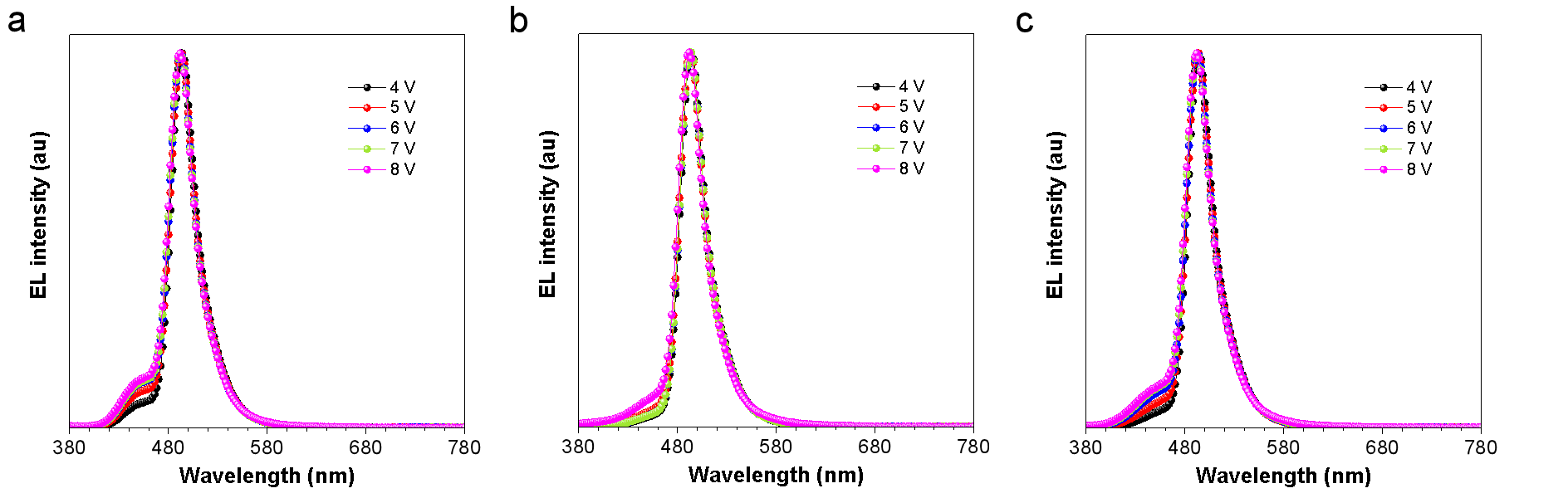


**Fig. S15** **The EL spectra of devices IS1‒IS3 at different voltages. a** The EL spectra of device IS1 at different voltages. **b** The EL spectra of device IS2 at different voltages. **c** The EL spectra of device IS3 at different voltages.


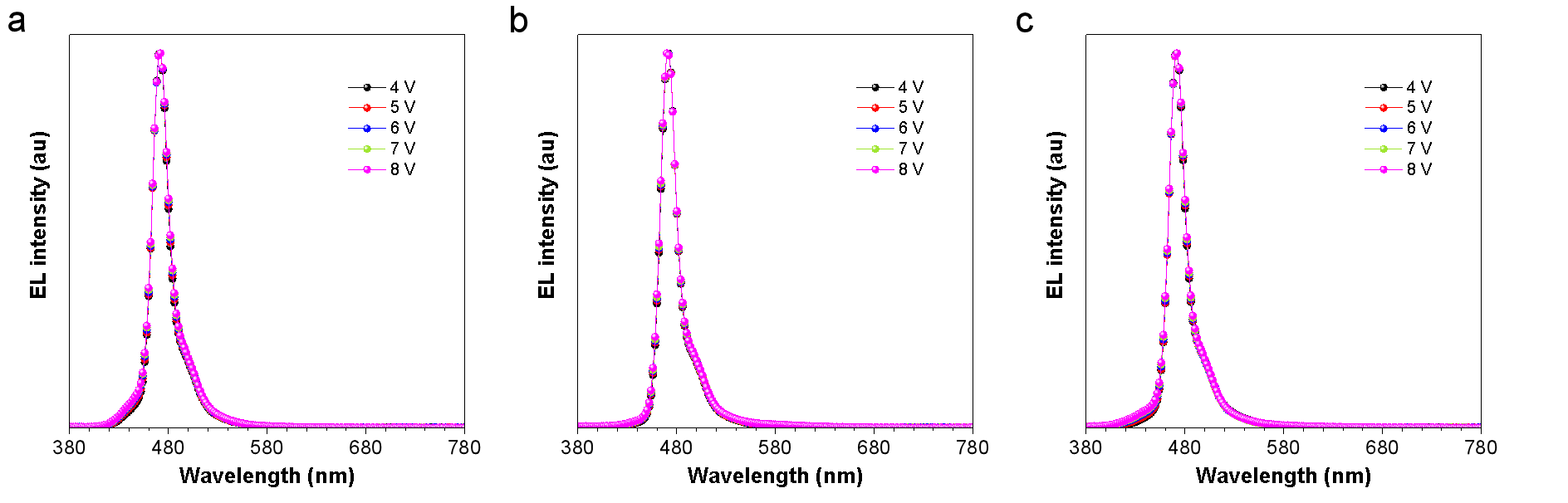


**Fig. S16** **The EL spectra of devices IS4‒IS6 at different voltages. a** The EL spectra of device IS4 at different voltages. **b** The EL spectra of device IS5 at different voltages. **c** The EL spectra of device IS6 at different voltages.


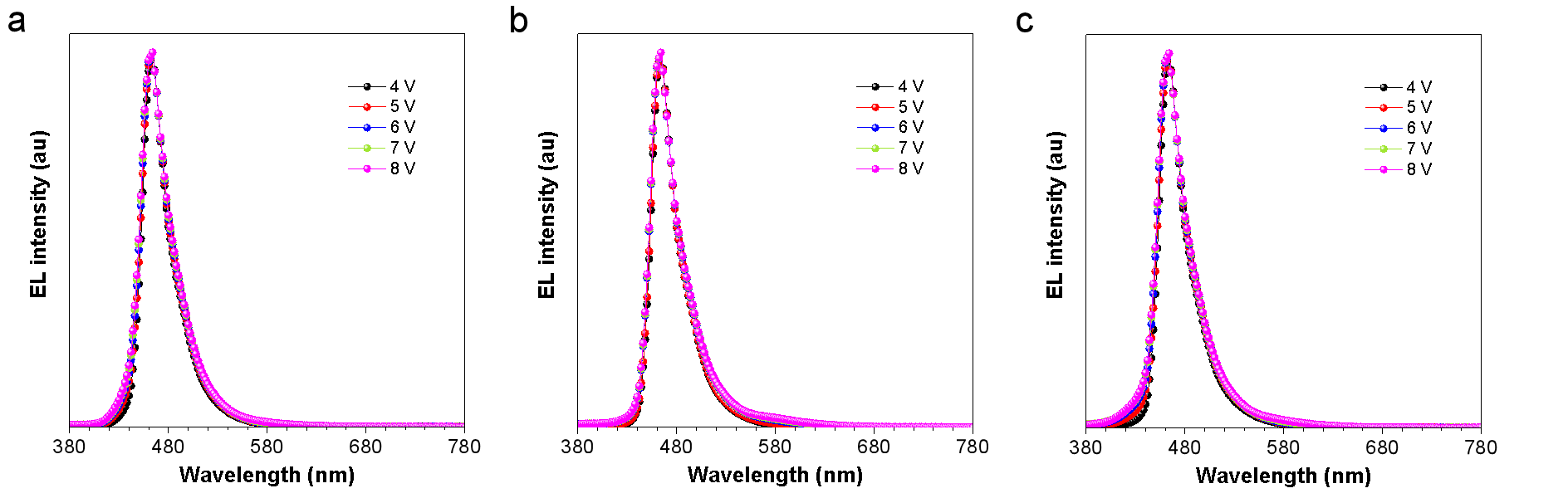


**Fig. S17** **The EL spectra of devices IS7‒IS9 at different voltages. a** The EL spectra of device IS7 at different voltages. **b** The EL spectra of device IS8 at different voltages. **c** The EL spectra of device IS9 at different voltages.


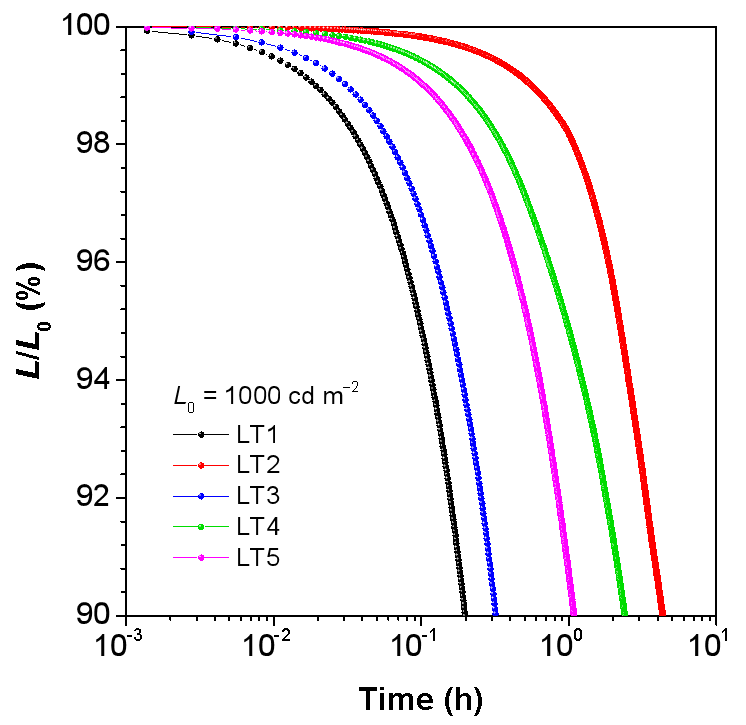


**Fig. S18** **Operational lifetimes of devices LT1‒LT5.**

**Table S1. EL performances of MR-TADF emitters and TADF sensitizers.**

| Device^a)^ | EML | λ_EL_  [nm] | *V*_on_  [V] | *L*_max_  [cd m^−2^] | EQE [%] | CIE (x, y) | FWHM  [nm] |
| --- | --- | --- | --- | --- | --- | --- | --- |
|  |  |  |  |  | Max/100 cd m^‒2^  /1000 cd m^‒2^ |  |  |
| － | 1 wt% BNCz-pTPA: mCBP | 492 | 3.6 | 26560 | 32.2/28.4/19.0 | (0.089, 0.387) | 28 |
| － | 1 wt% *v*-DABNA: mCBP | 470 | 3.8 | 23330 | 19.0/15.6/12.4 | (0.123, 0.121) | 19 |
| － | 1 wt% *t*-DABNA: mCBP | 464 | 3.8 | 7270 | 14.1/7.5/2.4 | (0.136, 0.112) | 27 |
| SB1 | 20 wt% TBCz-XT: mCBP | 446 | 2.8 | 13060 | 17.8/14.5/10.4 | (0.150, 0.099) | － |
| SB2 | 20 wt% TBCz-XT: PPF | 464 | 3.0 | 18080 | 30.1/27.6/20.9 | (0.150, 0.182) | － |
| SB3 | 20 wt% DMAC-DPS: mCBP | 470 | 3.0 | 15360 | 19.9/19.6/16.0 | (0.162, 0.241) | － |
| SB4 | 20 wt% DMAC-DPS: PPF | 474 | 2.8 | 14430 | 29.8/26.4/21.9 | (0.165, 0.240) | － |
| SB5 | 10 wt% PPCzTrz: mCBP | 454 | 3.2 | 28490 | 20.2/15.6/12.0 | (0.156, 0.156) | － |
| SB6 | 10 wt% PPCzTrz: PPF | 470 | 3.2 | 20170 | 34.1/25.9/14.8 | (0.166, 0.232) | － |

1. Device structure: ITO/HATCN (5 nm)/TAPC (50 nm)/TCTA (5 nm)/mCBP (5 nm)/EML (20 nm)/PPF (5 nm)/TmPyPB (40 nm)/LiF (1 nm)/Al (120 nm). Abbreviations: *V*_on_ = turn-on voltage at 1 cd m^−2^; *L*_max_ = maximum luminance; EQE = external quantum efficiency; *λ*_EL_ = EL peak; CIE = Commission Internationale de l’Eclairage coordinates; FWHM = full width at half maximum.

**Table S2. Parameters for Förster energy transfer radius calculation of different TADF sensitizers.**

| TADF sensitizer | MR-TADF emitter | Host | *Ф*_PL_ [%] | *J* [× 10^-14^ ] | *R*_0_ [nm] |
| --- | --- | --- | --- | --- | --- |
| TBCz-XT | BNCz-pTPA | PPF | 94 | 15.11 | 4.47 |
|  |  | mCBP | 64 | 16.13 | 4.23 |
|  | *v*-DABNA | PPF | 94 | 4.93 | 3.70 |
|  |  | mCBP | 64 | 7.10 | 3.69 |
|  | *t*-DABNA | PPF | 94 | 2.40 | 3.29 |
|  |  | mCBP | 64 | 4.43 | 3.41 |
| DMAC-DPS | BNCz-pTPA | PPF | 90 | 14.27 | 4.39 |
|  |  | mCBP | 70 | 15.08 | 4.25 |
|  | *v*-DABNA | PPF | 90 | 5.11 | 3.70 |
|  |  | mCBP | 70 | 5.58 | 3.60 |
|  | *t*-DABNA | PPF | 90 | 2.85 | 3.36 |
|  |  | mCBP | 70 | 3.15 | 3.27 |
| PPCzTrz | BNCz-pTPA | PPF | 93 | 14.25 | 4.41 |
|  |  | mCBP | 73 | 15.22 | 4.29 |
|  | *v*-DABNA | PPF | 93 | 5.48 | 3.76 |
|  |  | mCBP | 73 | 7.08 | 3.77 |
|  | *t*-DABNA | PPF | 93 | 3.18 | 3.44 |
|  |  | mCBP | 73 | 4.86 | 3.54 |

^a)^ Abbreviations: *Ф*_PL_ = PL quantum yield of the host in the absence of guest; *J* = the spectral overlap integral between host PL and guest absorption; *R*_0_ = Förster energy transfer radius.

**Table S3. EL performances of the hyperfluorescence devices using exciplex host.**

| Device^a)^ | λ_EL_  [nm] | *V*_on_  [V] | *L*_max_  [cd m^−2^] | CE  [cd A^−1^] | PE  [lm W^−1^] | EQE [%] | CIE (x, y) | FWHM  [nm] |
| --- | --- | --- | --- | --- | --- | --- | --- | --- |
|  |  |  |  |  |  | Max/100 cd m^‒2^/1000 cd m^‒2^ |  |  |
| S10 | 492 | 2.8 | 45310 | 55.7 | 62.5 | 32.0/28.0/21.0 | (0.107, 0.333) | 30 |
| S11 | 492 | 3.0 | 38860 | 57.9 | 60.6 | 30.8/27.5/20.8 | (0.106, 0.386) | 30 |
| S12 | 492 | 2.8 | 36180 | 61.4 | 68.9 | 33.7/27.6/18.5 | (0.108, 0.362) | 30 |

^a)^ Abbreviations: *V*_on_ = turn-on voltage at 1 cd m^−2^; *L*_max_ = maximum luminance; CE/PE/EQE = current efficiency/power efficiency/external quantum efficiency; *λ*_EL_ = EL peak; CIE = Commission Internationale de l’Eclairage coordinates; FWHM = full width at half maximum.

**References**

1. Jeon, W. S. et al. Ideal host and guest system in phosphorescent OLEDs. *Organic Electronics* **10**, 240‒246 (2009).
2. Liu, H. et al. Achieving high electroluminescence efficiency and high color rendering index for all-fluorescent white OLEDs based on an out-of-phase sensitizing system. *Advanced Functional Materials* **31**, 2103273 (2021).
3. Lin, G. W. et al. Improving electron mobility of tetraphenylethene-based AIEgens to fabricate nondoped organic light-emitting diodes with remarkably high luminance and efficiency. *ACS Applied Materials & Interfaces* **8**, 16799‒16808 (2016).
